# Editorial: Neonatal procedural pain management (vol II)

**DOI:** 10.3389/fped.2023.1270055

**Published:** 2023-08-31

**Authors:** Paola Lago

**Affiliations:** Neonatal Intensive Care Unit, Department of Critical Care, Cà Foncello regional Hospital, Treviso, Italy

**Keywords:** neonatal pain, neonatal pain management, procedural pain management, pain measurement, analgesia, sedation, neonatal palliative care

**Editorial on the Research Topic** Neonatal procedural pain management (vol II)

Procedural pain in newborns is one of the most challenging tasks for neonatologists and nurses. The adverse health consequences of untreated pain in vulnerable newborns is already well known, especially in preterm infants, and uncontrolled nociceptive inputs may alter nervous-system development in a critical phase of growth. However, the possible neurotoxicity of drugs in the developing nervous system are a concern, and it is necessary to balance between the efficacy and safety of pharmacological and non-pharmacological interventions.

There are many recommendations for treating neonatal pain, which point out the importance of identification, evaluation, and monitoring of pain, and many pain scales for newborns have been validated. However, there is still no gold standard. Particularly during painful procedures, pain assessment is difficult and many times neglected in clinical practice (1).

Llerena et al. systematically reviewed 13 neonatal pain scales. All of them have strengths and limitations, but none seem to comprehensively address different aspects of neonatal pain. Inductive machine learning algorithms can be used to recognize various expressions of pain by integrating physiological, behavioral, and electrophysiological parameters, which are used to assess EEG cortical activity and tone changes in the parasympathetic and sympathetic nervous system in multiple dimensions. Perhaps, such algorithms could be a valid tool to improve our capacity to recognize and identify pain in newborns in the future.

It is also important to clearly define tools for acute, procedural, prolonged, and chronic pain, as well as for measuring the analgesic efficacy and sedation conditions. Together with inter-rater reliability, intra-rater reliability, and validity, such tools should also have good clinical applicability. Only in this way can we correctly recognize and manage neonatal pain.

On behalf of the *Société Française de Néonatalogie*, Durrmeyer et al. made an important contribution to research in this collection. Neonatal laryngoscopy for tracheal intubation or surfactant administration is a painful procedure that is often performed in the neonatal intensive care unit (NICU). Since 2010, the American Academy of Pediatrics has stated, “Except for emergent intubation during resuscitation either in the delivery room or after acute deterioration or critical illness at a later age, premedication should be used for all endotracheal intubations in newborns” (2). Nevertheless, in clinical practice, there is still wide variability in the use of premedication.

In Italy, the routine use of premedication for non-emergency tracheal intubation was reported by 40% of NICUs interviewed in a survey, but the results are not exactly recent (3). Many studies have been demonstrated that the use of analgesia and sedation (AS) for intubation of newborns significantly improves intubating conditions, decreases the time and number of attempts needed to complete the maneuver, and minimizes airway trauma. Thus, the authors strongly recommend the use of analgosedation.

On the other hand, there is still insufficient evidence on the best sedative and analgesic regimen that should be used. Obviously, a laryngoscopy performed to start mechanical ventilation is different from one that is performed for INSURE (INtubation-SURfactant-Extubation) or LISA (Less Invasive Surfactant Administration) as the infant's respiratory drive should be preserved in the latter situation. Evidence-based practice guidance has been provided for tracheal intubation or LISA administration and laryngeal mask insertion using GRADE methods to qualify the level of evidence and the grade of recommendation. The proposed algorithm certainly has high clinical utility and confirms what has been stated for more than 10 years since the AAP's recommendations (2). These circumstances contribute to the difficulty in performing clinical studies on drugs for neonates and preterm infants.

The increasingly widespread use of ultrasound-guided central catheters makes the work by D'Andrea et al. very timely and interesting. Although their study was retrospective, it tries to provide a practical answer in regard to what is a good approach when performing this painful procedure in a possibly motionless patient, while minimizing the risk of complications. They showed that the combination of fentanyl and ketamine seems to be effective for the successful insertion of a centrally inserted central catheter without major side effects. It is important emphasize that the skill and experience of the operator are also very important for the success of the procedure as well as a good sedation and analgesia.

Nakhleh-Philippe et al. point out the importance of an adequate AS regimen during therapeutic hypothermia (TH), which is the standard of care for newborns with moderate to severe hypoxic-ischemic encephalopathy. According to a recent survey and retrospective analysis, most Italian NICUs use AS during TH (4), as in France. The authors evaluated the impact of AS on the comfort of hypothermic infants and follow-up in terms of abnormal neurological evaluations. They used a combination of fentanyl and midazolam to guarantee good comfort and modulated the doses according with the AS evaluation. However, excessive sedation was found in more than 57% of infants. This is because hypothermia and the presence of multi-organ dysfunction make drugs' metabolism and individual response unpredictable.

It is important to keep on researching models of pharmacokinetics and pharmacodynamics in this clinical setting in order to optimize analgosedation in the asphyxiated infant treated with therapeutic hypothermia.

This research topic has offered a unique opportunity to explore recent advances in assessing and treating neonatal pain. Alleviating pain is essential for not only supporting the wellbeing of newborns, but also protecting their motor, cognitive, and psychological development.
